# Becoming a podiatrist: an exploration of the practices and processes which underpin the acquisition of a professional identity

**DOI:** 10.1186/s13047-023-00652-w

**Published:** 2023-08-19

**Authors:** Jane Tobbell, Peter Roberts

**Affiliations:** 1 Department of Psychology, University of Huddersfield, Queensgate, Huddersfield, HD1 3DH UK; 2 University of Huddersfield, Queensgate, Huddersfield, HD1 3DH UK

**Keywords:** Podiatry, Identity, Imagination, Engagement, Alignment, Community-of-practice, Ethnography

## Abstract

**Background:**

Undergraduate podiatry degrees are designed to enable students to become professional podiatrists. To be successful students must manage academic and practical activity to ultimately acquire a professional identity. Little is known about the practices and processes which underpin the acquisition of a professional podiatry identity. It is the aim of this paper to begin to address this absence of knowledge. Community of Practice theory, arguably the dominant contemporary learning theory, represents identity shift as an interaction of imagination, engagement and alignment which enables students to successfully participate in higher education, and ultimately, the professional context. This success is underpinned through assisting students to develop an enabling identity in their learning and doing.

**Methods:**

Here we present findings that emerged from a yearlong ethnography in a successful higher education podiatry department. The project followed students and staff in the classroom and the clinic and explored their experiences through interviews.

**Results:**

The findings suggest that the journey to professional identity is facilitated through meaningful learning relationships between staff and students and clarity around professional practices. Here we discuss how those relationships form and enable undergraduates to become podiatrists.

**Conclusions:**

Our findings offer a model for the transition from student to professional and highlight the importance of relationship and experience in becoming a podiatrist. There is a paucity of research around not only podiatry but also other allied health professions around this topic and given the increasing emphasis around employability skills in HE, more research in a range of contexts is needed.

## Background

The acquisition of professional identity is a socio-cultural process which requires that putative professionals participate in the valued practices which define their chosen occupation [1, 2]. Through presence, relationship and activity, student professionals acquire the ability to perform the necessary skills, acquire the necessary knowledge and understand the explicit and implicit values which characterise the profession. The profession, in socio-cultural terms, can be thought of as a community of practice. It is defined by established and shared goals which are achieved through collaborative practice.

UK podiatrists operate under the regulation of the Health and Care Professions Council (HCPC). The title “podiatrist” is protected within legislation and registrants are bound by a shared code of conduct, performance and ethics [3]. Through these standards the parameters of practice, and legal scope of work as a podiatrist are made transparent. Contrasts between ideal, abstract conceptions and real performance of practice and the status of the profession have been identified as contemporary problems in podiatry [4–7]. Despite this, the core shared identity of podiatry remains that of a clinician responsible for the assessment, diagnosis and management of conditions affecting the lower limb and foot [7].

The process of acquiring the professional identity is not, of course, as straightforward and simple as it seems on paper. The community of practice (CoP) cannot be thought of as a neutral context for incoming members. It is a context which has developed over many years and through a myriad of relationships; inclusion in the community is an intricate process of belonging. The goal of professional education and training is to assist in the formation of a trajectory which allows students to invest their present in their future as a podiatrist [8]. To date the process by which this happens in undergraduate podiatry education in the UK is largely unexplored. Here we present findings from one podiatry department and offer an analysis of identity acquisition which we hope will encourage more research to enable effective education and thus better podiatrists.

**Fig. 1:**
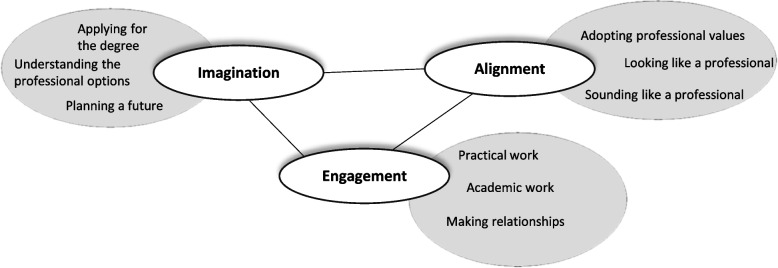
Modes of belonging invocational students (adapted from Wenger 1978, p174)

The process of belonging is underpinned by three different modes which interact in complex and non-predictable ways, resulting in identity shifts. Figure 1 depicts the three modes of belonging: imagination, alignment and engagement. It also suggests practices around these modes which are necessary for identity shift. Imagination allows the student to represent what might be, to appreciate what success might bring. In engagement mode the student is actually performing the practices which pave the way to success. These work in concert with alignment where a student consciously or unconsciously identifies themselves with the ethics, standards and beliefs of the profession.

The notion of practice is an important one. From the perspective of community of practice theory it can be understood as representing the meanings which underpin the functioning of any community – in a podiatry sense the profession has ways of being that are defined by language, actions and the way they look and behave.

Podiatry has a language drawn from various branches of medicine, dermatology, neurology, endocrinology, rheumatology and orthopaedics in particular. The clinical environments of podiatry are often similar in essentials, and clinicians, despite much inter-professional working tend to operate independently with patients in one-to-one scenarios. But a practice is more than this, it is also that which defines a community. An example in podiatry is the unified focus on preventing mortality and promoting mobility for patients with diabetes mellitus. In the sphere of diabetic foot care, core podiatry skills such as wound debridement, patient education and musculoskeletal intervention are interwoven with more specialised activities such as independent prescribing (or the use of prescription only medicines), clinical investigations, diagnostic imaging and podiatric surgery. With the specialty of diabetic foot care also underpinned by research, continued professional development and education, it encapsulates the whole community of podiatry practice.

A practice is a dynamic concept, that which defines podiatry has changed and will change over time. The shift from core podiatry to the utilisation of more advanced skills has already seen the implementation of podiatric surgery and independent prescribing [9, 10]. In addition, the profession has established a position in the centre of a wider foot health workforce whereby core requirements can now be delegated more readily to assistant grades [11]. These shifts are not without challenge in terms of the boundaries with other professional groups [12] however, the movement has been towards establishing an identity of practice that extends beyond the foot whilst retaining the commonality of the COP [7]. The aim of podiatry undergraduate education is to prepare students to enter this profession in readiness to adopt the podiatry identity whilst having the agency to shift with and challenge the status quo.

Equally important as practice, is the notion of participation. There are many different types of participation from full participation, where the community member changes and is changed by practice, to non-participation, where membership of the community is peripheral and non-contributory. How a student participates is a key part of understanding their identity trajectory [13]. In order to promote the process of belonging and the concomitant identity shift, certain practices are more likely to enable participation and so allow for the productive interaction of the modes of belonging.

**Fig. 2:**
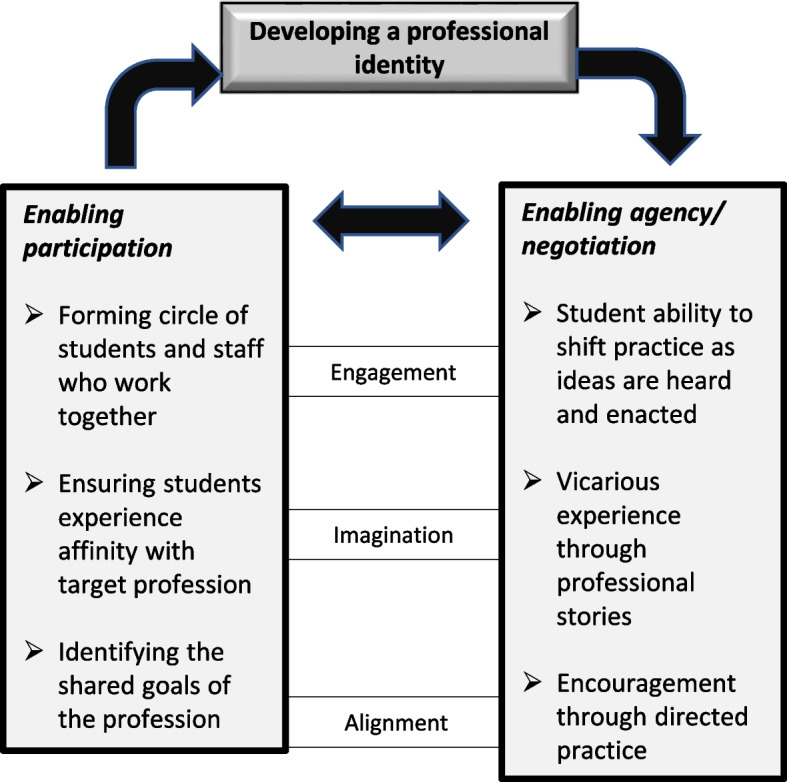
A model of the process of developing a professional identity (adapted from Wenger 1978, p190)

In Fig. 2, we outline the process of the development of a professional identity by showing the types of practice which might enable engagement, imagination and alignment to work together to stimulate participation and simultaneously enable the emergence of agency (we have renamed Wenger’s (1998) process of negotiation as agency [2]. Agency, the ability or sensation that a student is in control of their participation and learning, is a fundamental element in identity formation [14]. It enables the student to understand what is required and what they need to do to meet those requirements. Without it there may be confusion and random behaviour. Where students can imagine what it is like to be a podiatrist and so engage in the practices which enable this whilst aligning themselves with the espoused professional values, there is more possibility of the emergence of an effective professional podiatrist identity.

Here we have identified practices from the learning literature and applied them to the study of podiatry. There is little research available which provides empirical support for these ideas. Indeed, there is a dearth of pedagogic literature pertaining to podiatry all told. Recent exceptions being bodies of work around clinical placement experiences and opportunities [15–17], as well as inter-professional teaching and learning [18]. It is the aim of this project to begin to address this gap and contribute to an understanding of how to construct successful learning environments in the study of podiatry in higher education institutions.

### Research aim

To understand the process of professional identity acquisition in undergraduate podiatry students in a UK university.

## Methodology

An ethnographic approach underpinned data collection and analysis. The flexible and pragmatic nature of ethnography enabled the researchers to experience the culture of learning in the first-year podiatry cohort. Multiple sources of data; observation, interviews and a focus group were combined to develop understanding and generate an explanation of the topic [19]. This project has been conducted and reported with the consolidated Criteria for Reporting Qualitative Studies (COREQ) 32-item checklist in mind [20].

### Ethics

Ethical approval was sought and gained through the University of Huddersfield Research Ethics and integrity committee in the School of Human and Health Sciences.

## Methods

This ethnographic opportunity was identified owing to the general trend of success over several years of the podiatry programme in question. Metrics such as high student satisfaction, high employment or further study rates and complimentary employer feedback suggested this as an appropriate setting. This is not to suggest that the setting described here is superior to other podiatry training routes but within higher education there was evidence of persistent success to warrant pedagogic exploration within this project. This was particularly the case when the podiatry programme was compared to other courses available within the same institution.

Purposive sampling sought to include participants and settings which could help illuminate the topic by their shared characteristics; namely learning or teaching podiatry. Potential participants were approached face-to-face in year one induction sessions and their consent to participate agreed. Observation of clinical and theoretical teaching sessions for first year podiatry students was the primary source of data. The cohort size was 44. Both researchers observed sessions independently with a total observation time in excess of twenty hours. Observation notes were made freehand and without a pre-designed schedule to allow notation of any and all activities occurring during teaching. A non-participatory approach meant the researchers were not involved in teaching but were able to observe and experience the environment and practices first hand [21].

Individual interviews and a focus group interview were held with academic staff and students. Three members of academic staff were recruited to the study with different levels of experience ranging from less than 2 years, 7 years and 19 years respectively. A piloted semi-structured interview scheduled was used with prompts to remain on topic but also opportunities for the participants to direct the flow of conversation. Two final year students participated in a group interview which, despite the disappointing recruitment figure, yielded rich data providing insight into their experience of the learning culture.

Observations and interviews were conducted by both JT and PR. Both researchers had previous experience of ethnographic research and qualitative data collection at post-doctoral level. PR is a male podiatrist and podiatry academic and JT is an internationally published female psychologist. They were both introduced to participants at the start of the project and participants were made aware of their professional backgrounds and research interests.

The researchers met regularly during data collection to share and discuss their impressions and initial analyses. Both used a reflexive approach, keen to minimise and understand the impact of their presence on the research environment. Impact was predominantly reduced through consistent presence in taught sessions which reduced the novelty value and thus any Hawthorne or reactive effect [22–24]. Reflective notes were kept following observations which also served to identify potential impact of the researcher’s presence.

### Theoretical analysis

This research is ontologically and epistemologically informed by socio-cultural theory. As such, a structured analysis was carried out where data were coded through the lens of Fig. 1, the imagination, engagement, alignment modes, adapted from Wenger’s community of practice theory (1978). Each researcher independently coded the data under the above headings and then through discussion the data were categorised into imagination, engagement and alignment and subsequently emergent meanings were explored. These findings were then combined to generate a model of the process of acquiring a podiatry identity.

### Findings

#### Imagination

In imagination, students are able to go beyond what is, and think about what could be. They are able to use their knowledge and experience and position themselves in the podiatry profession. Clearly, they have already activated their imaginations in identifying and applying for the programme but they will need a richer picture to develop this. Once the student feels they belong to the professional community of practice, shared meanings are acquired which facilitate imagination [2]. Masika and Jones [25] note that part of the process of learning involves belonging which requires sustained engagement between tutors and students. In this research, tutors were focussed on enabling students to develop this sense of belonging:

*“They just need to know they belong. Which is easier on a small course like ours.”*

Tutor 1 interview

*“If you can be open and friendly, or appear to be friendly, then they are willing to ask questions.”*

Tutor 2 interview

Final year students indicate that this embracing culture underpinned their journey and talked about how their sense of who they are shifted as a result of staff activity:

*How far I’ve come and how confident we are. When I started, I was like this shy little girl … I really struggled with social anxiety… to speak with patients and staff.*

Student focus group

*Very often in our lectures, there’s the humour, their personal examples when they were students or some interesting cases when they were practicing, working in the NHS… they bring these life experiences, this sort of levels you. We are colleagues, they are our teachers but we are colleagues.*

Student focus group

Through a process of open exchange, imaginations are liberated, students are enabled to shift from student identity to colleague identity. Andersson and Köpsén [26], in their work with vocational education teachers, note that organisations need to support the interactive processes of imagination, engagement and alignment in order to assist the acquisition of new identities. In terms of becoming a podiatrist, it makes sense that students need the information about what a podiatrist is but also the psychological confidence to allow themselves to dream. Self-efficacy theory [27] posits that imagination is enabled by confidence around performance. Students who believe they can be successful are more likely to position themselves along trajectories of success. This demonstrates the connection between imagination and engagement and also highlights that facilitating imagination is not just about telling stories about podiatry. It is also about connecting students to those stories.

Observational data demonstrate the simultaneous emphasis on being a podiatrist from the outset and the invitations to students to become a podiatrist. Notes from the first day on the programme reveal this. In the first hour of their university career, they are shown the tools of the trade:

*The tutor shows picture of the podiatry pack which they will be coming on Friday to collect. She goes through the contents, asking people to identify each of the items. Some students know what everything is, others don’t.*

The tutor invites students to be part of the profession. She introduces, with pictures, the Royal College of Podiatry and invites students to join and participate:

*Talks about Royal College of Podiatry – “You don’t have to join but it’s like our union.” She flags up the upcoming conference. “If you go to that we give you a special dispensation from clinic on the Thursday as you will learn so much. £59 for students.”*

In teaching sessions constant reference is made to the practice of podiatry and students are given the information to imagine their futures:

*The tutor, when presenting clinical material, talks about clinics and patients they will see. Constantly showing how necessary knowledge is acquired in this lecture session and how this applies to practice in the clinic.*

*The tutor talks about plantar fasciitis “You’re going to learn about this and be able to pay your mortgage off by managing it.”*

The acquisition of knowledge is positioned alongside the process of becoming a professional. Students are given the ability to imagine how their learning in the classroom will eventually be rewarded through professional activity. Whilst students and tutors do not explicitly invoke ‘imagination’ as a goal, the facilitation of it is embedded in the practices of the programme as evidenced by the data above. Imagination is a necessary part of the acquisition of a professional identity and so both teachers and students should be encouraged in its invocation.

### Engagement

There are debates around the notion of engagement in education but here we are specifically concerned with understanding engagement as part of the process of becoming a professional podiatrist. An emergent meaning from this data was that engagement was enacted in a complex way. It involved opportunities for student agency together with enabling learning relationships. Tobbell and O’Donnell [28] have argued that effective learning relationships underpin successful transitions and that the emergence of these is situated in the context but always features meaningful student – staff interaction and the acknowledgement of individual student experience. On this programme student feelings and emotions were acknowledged, permitted and normalised.

The observational data reveal that students were invited to participate from the beginning. Field notes from the induction sessions:

*The tutor introduces herself and outlines her roles in the department. She acknowledges that at this moment people are feeling a mixture of difficult emotions. She sets up a Mentimeter – lots of emotions such as confused, inspired, overwhelmed (mix of hard and good). Students invited to complete the Mentimeter on their phones. Quite a lot of talking between students.*

*The tutor reveals results and talks them through ‘If you are feeling worried and anxious, you’re not on your own. Lots of people are feeling those things.’*

This permission to feel vulnerable emerges from fieldnotes in teaching sessions. After just two weeks of teaching students are expected to engage in formative tasks and are asked about it in class but also given permission to be learners:

*The tutor, referring to an identification task on the VLE from last week where they have to label a diagram from memory: “How are you finding the task? Are you finding you remember more than you think?’ ‘Don’t be hard on yourselves, its only week 2.’*

*She goes on to talk about the contact she’s had from students. She refers to emails she has received and praises this “I can tell you are doing work outside.” Some of the emails have expressed anxiety around performance in the module exam, she offers lots of reassurance about anyone feeling overwhelmed.*

*The support is not just verbal, the tutor goes over the assessments in the module “I’ve put together some formative questions … you can see what you’ll be asked in the exam.” She talks about how she will provide extra resources on the VLE (Virtual Learning Environment).*

The process of engagement here is enabled through the tutors demonstrating an understanding of the challenging emotions that surround early participation in a new environment but also by practice, by listening to the students and responding to their expressed needs. This approach to teaching is reflected in staff interview responses:

*“There’s been lots of times when you’ve helped students but it’s not about their work, it’s about their personal life.”*

Tutor 1 interview

*“If you can be open and friendly, or appear to be friendly, then they are willing to ask questions.”*

Tutor 1 interview

*“It’s not so much be their best friend but know about them, know their life and understand them, then you can make a difference.”*

Tutor 2 interview

*“Because of the cultural differences when she is at home there are lots of people around and she has extra responsibilities. So we sat together and devised a timetable of her studies that she would need to be in uni for so that when she would be away from those responsibilities at home.”*

Tutor 3 interview

Importantly the engagement is not just staff – student. There is a sense of camaraderie amongst students:

*[In clinic] … that’s where most of the help comes from each other. The tutors are dividing their time … so we ask each other ... You say I experienced this last week, I had something similar so can I help you? … we have confidence in each other that we will help. … [It’s developed] over time, spending a lot of time together.*

Student focus group

This is encouraged from the beginning through a buddy system, where final year students support other students in the clinic. This is valued as a learning opportunity:

*With the buddy roles, you’ll see year 3s helping year 1s, we know what it’s like to be in that position so we can help.*

*It sort of took you back to year one which you might not have gone over, takes you back to basics… I learn from that, relaying that to someone else, spieling that off to someone else helps you learn.’*

Student focus group

Students are given multiple opportunities to enact the practices which define their progression to a professional identity. They are supported in their initial struggles to participate in podiatric theory and its application. As they engage in more complex ways, the students become the source of knowledge and skills and can share these with new students. Through such a process they become more aware of their accomplishments.

### Alignment

Through the process of engagement students are made aware of the essential meanings and practices which define the profession of podiatry. They are required to participate in many of these practices and as time passes and participation increases in complexity, students become more defined by the valued practices and so move towards a professional identity.

From the outset students on this programme are made aware of their responsibilities and how they will be held accountable. The fieldnotes reveal:

From induction students are made aware of their professional accountabilities:

*The tutor introduces the HCPC. She explains that if there are any practice issues, if students do the wrong thing, there will be a hearing. It will be fair but if they do contravene professional practice expectations they will be off the course. Said in a very straightforward way, not threatening but clear.*

Students are also made aware of the university accountabilities:

*Moves on to academic expectations. “Your engagement on the VLE is monitored. If you’re doing ok you’ll be left alone, if you’re not doing well we will monitor you. It notes how many times you enter and how long you are on for.”*

*Goes through attendance expectations. “You need 100% attendance at clinics.” Explains why. “You need 1000 hours to qualify, if you miss clinics you will have to make up hours to get qualified. If you don’t attend, you need to contact admin to explain why.”*

The rules for participation in the clinic were also clearly articulated:

Further on in the session, the tutor provides clear direction about personal presentation in the clinic:

*Moves on to how to present themselves in the clinic. She explains that there are regulations about appearance. For each regulation she gives reasons and examples.*

*No sleeves below the elbow. Disposable sleeves available if culturally students don’t expose arms.*

*No heavy jewellery.*

*Footwear, plain black, low heels – you are foot models. No trainers, no open toed shoes. Shows picture of a scalpel in the foot. Some discussion about leather and vegan leather with vegan students.*

*Vaccinations are mandatory – the tutor asks students why this is? There is some discussion and then she explains that they might be working with immunocompromised (explains that means don’t have much immunity) clients.*

*If you can’t make a vaccination appointment, please tell them – there are a lot of health students, so they are very busy. “If you don’t complete all your vaccinations then you are off the course.”*

But alignment is not just mandated. The practices are also inculcated through relationship, the provision of additional support and scheduled teaching sessions:

*“I’ve arranged for whoever is with her on clinic to sit and go through personal feedback on where she has done well and pointers for where she could improve for the next clinic”.*

Tutor 3 interview

*“We try and help our students a lot with revision tips and that is often a place where they come and speak to you about.”*

Tutor 1 interview

In teaching observations, there is evidence of supporting the process of acquiring the language of the profession:

*The tutor talks about the nail and highlights that this will feature today and in future sessions. She says “I spoke last week about the importance of linking this module to your clinical practice module. The more experience you have the easier these terms will become.”*

The final year students acknowledge this process of alignment:

*Yesterday, I was looking round the class and remembering the first year and how we’ve changed. How we sound now, how professional, we’ve grown a lot. What was good was the structured ... how structured the learning was that got us where we are now. How theory led to practice … it was nice in the first year how we learned in the theoretical lecture and how we used that in the clinic.*

Student focus group

They also reveal how the clinic enables the professional identity:

*I like when lecturers treat you as an equal, even though we’re not that experienced. I think when they ask your opinion … if in clinic we have difficult cases and we all work together to try and figure out what is the problem.*

Student focus group

The process of alignment then is a combination of explicit instruction and implicit practice. Students are told how to look like a podiatrist, through activity in the clinic they learn how to behave like a podiatrist and this is enabled by participation in classroom learning and assessment.

## Discussion

### Modes of belonging

**Fig. 3:**
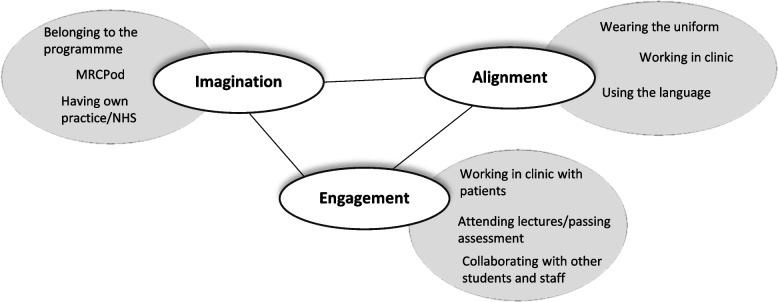
Modes of belonging in podiatry students (adapted from Wenger 1978, p174)

In the literature review the interactive processes of imagination, engagement and alignment were described with reference to vocational education in general. We can now begin to populate these modes to represent the activities which underpin the development of a podiatrist identity (Fig. 3).

The figure seeks to present a summary of the emergent findings from this research and provide examples grounded in podiatry education and practice to assist in understanding what activity defines the modes of belonging in podiatry specifically. Whilst we have separated the three modes of belonging in this analysis, this is an artificial separation. The same activity can stimulate the imagination, promote engagement and inculcate podiatric practice. It may also be that that which stimulates imagination in one person, fails to in another. In this research we have sought to provide some examples from our particular context, rather than offer a definitive truth. However, we would argue that participation in the modes above are a necessary contribution to the process of identity development.

### The process of developing a professional podiatrist identity

**Fig. 4:**
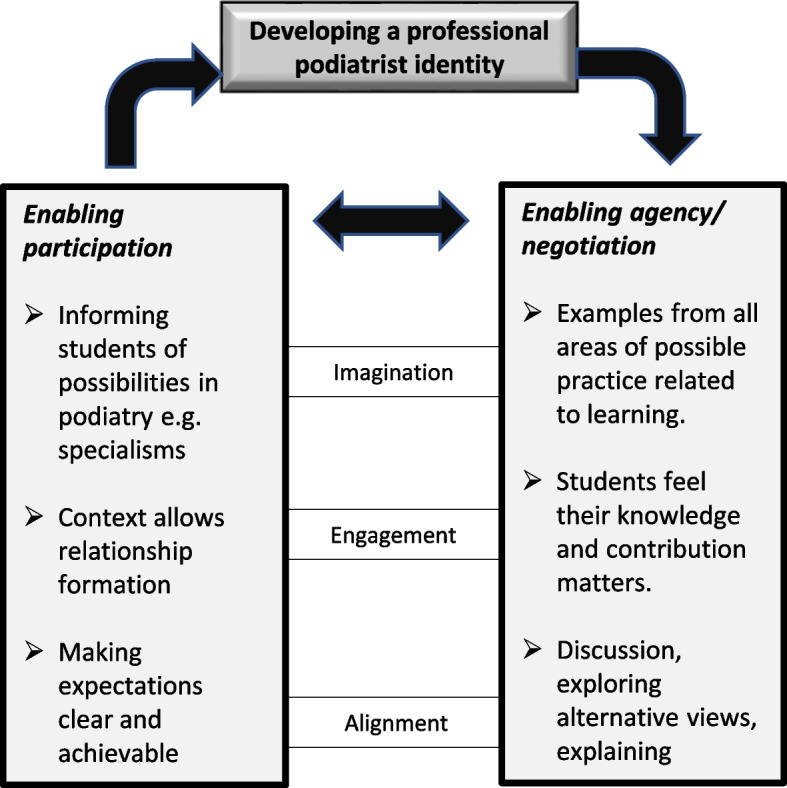
A model of the process of developing a professional podiatrist identity (adapted from Wenger 1978, p190)

We were fundamentally interested in the process of identity development which can inform educational development in the profession. Figure 4 builds on the more general representation of identity development in Fig. 2 and makes specific reference to participation and agency in podiatry education. We would argue that enabling participation and student agency are necessary in the effective development of a professional podiatrist identity but acknowledge that the ways of doing this are many and varied and emerge in the context.

### Study limitations

This qualitative study was conducted with the aim to understand the process of professional identity acquisition in undergraduate podiatry students in a UK university. The intention is not for generalisability but to contribute understanding by consideration of our specific context. As such the low recruitment numbers for the student focus group, whilst disappointing, has not detracted from richness of our findings.

### Concluding comments

Learning is about the frequent and consistent interactions that characterise the every-day culture of a context rather than one-off events unrepresentative of other experience. Our research is about becoming a podiatrist rather than re-imagining the profession. Given the imperative for higher education departments to work in partnership with allied health professions to train and educate professionals, an understanding of the process is useful to the HE staff, the students and the practice partners. This research featured a successful department, one with excellent employability and satisfaction data, it is therefore a credible context for exploring enabling teaching and learning activity in podiatry. Our findings offer a model for the transition from student to professional and highlight the importance of relationship and experience in becoming a podiatrist. There is a paucity of research around not only podiatry but also other allied health professions around this topic and given the increasing emphasis around employability skills in HE, more research in a range of contexts is needed. Further research, in a range of podiatry education contexts, could provide a richer picture of how a professional identity is acquired. This could include apprenticeship programmes, post-graduate conversion courses as well as traditional university undergraduate departments. It would also be useful to conduct longitudinal research which follows podiatrists through the first years of their professional lives.

## Data Availability

Not applicable.
